# Selenium Content in the Gonads of Healthy Cats (*Felis catus*) and Cats with Impaired Homeostasis from the Warsaw Area (Poland)

**DOI:** 10.3390/ani14030440

**Published:** 2024-01-29

**Authors:** Ewa M. Skibniewska, Michał Skibniewski

**Affiliations:** 1 Department of Biology of Animal Environment, Institute of Animal Science, Warsaw University of Life Sciences, Ciszewskiego Street 8, 02-786 Warsaw, Poland; 2 Department of Morphological Sciences, Institute of Veterinary Medicine, Warsaw University of Life Sciences, Nowoursynowska Street 159, 02-776 Warsaw, Poland; michal_skibniewski@sggw.edu.pl

**Keywords:** selenium, cats, testicles, ovaries, health, homeostasis

## Abstract

**Simple Summary:**

The purpose of the ongoing research was to determine the Se content of the gonads of male and female domestic cats and to analyze the relationship between Se levels and obesity and mammary gland tumors. It was found that the gonads of males have a higher Se content compared to females. In addition, in female individuals its content decreases with age. With regard to health status, cats can be ranked in the following descending order: healthy individuals, obese cats, individuals with mammary gland tumors. A marked decrease in its content with the age of animals may be the cause of increased cancer incidence in older animals due to the weakening of mechanisms that protect against the effects of peroxidation of cellular structures and immune system function.

**Abstract:**

The aim of the present study was to evaluate selenium content in the gonads of healthy cats and those with impaired homeostasis with the use of fluorescence spectroscopy. Higher concentrations of selenium were found in the gonads of the male domestic cats studied. The average content of this element in the testes of healthy males was: 0.59 mg·kg^−1^ while 0.47 mg·kg^−1^ wet weight was recorded in the ovaries of females. In the case of individuals with impaired homeostasis, higher values of selenium were found in the ovaries of obese females (0.40 mg·kg^−1^), while in the case of females with diagnosed malignancy of the mammary gland, the average values were lower and amounted to 0.31 mg·kg^−1^ wet weight of tissue. On the basis of statistical analysis, significant differences were found according to sex, age, and disturbed homeostasis of the individuals studied. An association was found between low Se in the gonads and obesity, as well as the presence of mammary gland neoplastic lesions. In addition, Se content was found to decrease with age in females, which can reduce resistance to oxidative stress.

## 1. Introduction

Selenium (Se) is a micronutrient essential for the proper functioning of the body, as the element is involved in many biological processes. Its role in maintaining homeostatic mechanisms is mainly due to its presence in selenium-dependent enzymes, which play a key role in metabolic processes. Se participates in antioxidant defense, endocrine system function, including thyroid hormone metabolism and DNA synthesis. Its adequate levels are also important for maintaining normal bone metabolism and immune system function. It is a component of glutathione peroxidase (GPx), and thioredoxin reductase (TrxRs) which are the main components of the enzymatic system of protection against peroxidation of cellular structures. Their main function is to neutralize reactive oxygen species in the intracellular and extracellular space [1,2,3]. Selenium also plays a key role in the functioning of the reproductive system in both males and females [4,5,6,7]. Among others, this element is essential for proper spermatogenesis. Testicular tissue contains high concentrations of selenium, mainly in the form of glutathione peroxidase 4 (GPX4) [4,8,9]. Irvine [10] suggests that increasing Se intake enhances the antioxidant activity of GPX and consequently improves male fertility. Selenium’s association with male fertility has been linked to its antioxidant activity, and its effect on the normal function of testicular duct cells responsible for normal sperm morphology [11]. Selenoprotein has been identified as an important component of the sperm cell membrane and the middle part of its tail, hence, there is a link between sperm motility and the degree of Se supply [12]. Spermatozoa contain a high concentration of polyunsaturated fatty acids (PUFA) in their phospholipids, which is an important risk factor for peroxidative damage to their cell membranes leading consequently to reduced male fertility. A complementary element to the action of Se is vitamin E, which, in combination with selenium, plays an essential role in protecting the lipid components of sperm cell structures against peroxidation. Accordingly, high levels of this element, necessary for the process of sperm production and maturation, are recorded in the testes and epididymides. Based on studies conducted on young boars, it was shown that the addition of 0.5 ppm of selenium to feed rations has a positive effect on semen quality, sperm motility, sperm concentration and morphology, as well as fertilization rate [13].

Contaminants in the food chain can have harmful effects on the ecosystem, but also on human and animal health [14]. Harmful substances can enter the body by inhalation, ingestion or directly through the skin, and accumulate in various organs including the gonads [15,16]. Long-term exposure to harmful environmental factors, leads to disruption of homeostasis. These processes occur at both the cellular and structural levels, causing harmful health effects in humans and animals. They include neoplasms, such as mammary gland cancer, which is one of the most common neoplastic diseases in cats. Selenium is known to exhibit anti-angiogenic effects and can inhibit the growth of malignant cells through cytotoxic effects [17,18]. Due to the inhibitory effect of Se on the growth of malignant cells, its levels in malignant epithelial mammary tissues in cats tended to be lower than in hyperplasia and dysplasia of the mammary gland [19]. In addition, as an antioxidant, it can reduce the toxicity of conventional chemotherapy to the body’s normal cells and modulate the oxidative stress induced by cell apoptosis through the regulation of caspase-3 and caspase-9 [17,18,19,20]. Therefore, it seems important to study the effects of selenium status on mammary gland tumors, which may clarify the mechanisms by which selenium can prevent tumorigenesis. Indeed, it has been found that in organic combinations as methylselenol, this element is involved in anticancer prevention [21,22]. Selenium’s mechanism of action involves preventing copper-mediated DNA damage, inhibiting cellular oxidation, promoting cell apoptosis, and regulating the immune system [23,24,25].

Feline mammary tumors (FMTs) are the third most common malignant tumors in female cats. FMT malignancy rates range from 80% to 90% [26,27]. Due to the hormone-dependence of mammary tumors in female cats, sterilization of individuals of this species before 6 months or 1 year of age can be considered as a form of cancer prevention. In this case, the age of the animals is one of the factors that significantly reduce the incidence of malignant mammary tumors [28].

Among other health disorders found in cats, there is the metabolic dysfunction associated with obesity, which is similar to the metabolic syndrome found in humans. The causes of obesity are not entirely obvious, as many factors influence its development. They can be divided into those that affect energy metabolism and those that affect energy intake and absorption. The prevalence of obesity in cats ranges from 6% to as high as 44%. Over the past 20–30 years, rising obesity rates in companion animals have occurred in parallel with the growing obesity epidemic in humans. As in humans, obesity in cats is also a multifactorial problem potentially related to other risk factors such as genes, gender, nutrition, physical activity, among others. There is a hypothesis that lack of exercise and poor diet not only predispose to higher levels of obesity in cats, but also exacerbate other health problems associated with being overweight [29,30,31,32]. Slingerland et al. [33] found that indoor cats have an increased risk of developing diabetes. In addition, Kienzle et al. [34] indicated that cat owners are less aware of alternative ways to interact with their cats, relative to dog owners, and consider feeding as the main point of contact with their pets. The authors also found a closer relationship occurring between overweight cats and their owners than between normal-weight cats and their owners, and a greater humanization of overweight cats than normal cats, as well as a potential role for overweight cats as a substitute for a companion [35].

Accordingly, the objectives of the study were formulated, which focused on the analysis of selenium content in the gonads of healthy cats and individuals with homeostatic disorders. The study attempts to answer the question of whether the age of cats is associated with changes in their selenium status, and whether obesity or mammary tumors in female individuals are related to the Se content of their gonads. To the authors’ knowledge, no attempt has been made to date to analyze the relationship of certain pathologies with selenium levels in the gonads of carnivores, on whose function depend, among others, the condition of the mammary gland and indirectly many metabolic processes. Therefore, it seems important to know about the above-mentioned relationships, which can help understand the importance of Se in maintaining normal homeostatic mechanisms.

## 2. Materials and Methods

### 2.1. Animals

The material for the study consisted of gonads collected from a total of 63 domestic cats (*Felis catus*). Gonads were collected during routinely performed sterilization and neutering procedures at veterinary clinics in Warsaw in 2020–2021. The study enrolled 35 healthy cats (young males n = 13, young females n = 6 and adult females n = 16) and females with disturbed homeostasis (n = 28)—individuals with diagnosed mammary malignant tumors of epithelial origin (n = 15), undergoing sterilization along with mastectomy and obese cats (n = 13). The healthy cats ranged in age from 6 months to 9 years and weighed from 1.5 to 6 kg, while the sick cats were aged from 3.5 to 13 years and weighed between 2.9 and 9 kg. Qualification of the animals’ status was based on a clinical examination at the time of qualification for surgery, during which an experienced clinician assessed the body condition score (BCS). All the cats were indoor animals. Ovaries and testes were collected whole and packed into polyethylene containers, which were cooled and transported to the laboratory for further processing. In preparation for selenium determination, organ preparation was performed to extract the appropriate samples. In the case of testicular tissue, the epididymis was separated and the white membrane (*tunica albuginea*) and the central part of the organ (*mediastinum testis*) were removed, as they contain the testicular network that constitutes only the sperm excretory pathways. Representative parenchymal samples were then taken from the central part of the testicular lobes. In the case of the ovaries, material was taken from the parenchymatous layer of the organ (*zona parenchymatosa*), known as the ovarian cortex after separation from its vascular layer (*zona vasculosa*) which is known as the ovarian core. Se content was determined only in tissue taken from the parenchymatous layer of the ovary.

Based on information obtained from the Second Local Ethical Committee in Warsaw, under current laws, research using animal tissues collected during routine prophylactic procedures and procedures performed for medical indications, treated as waste tissue, does not require additional permits.

### 2.2. Chemical Analyses

The test material was stored in a freezer at −20 °C until laboratory analyses. Selenium content was determined by fluorescence spectroscopy using a Shimadzu RF-5001 PC spectrophotofluorometer. The measurement was carried out with a fluorescence emission wavelength of 518 nm and an excitation wavelength of 376 nm. The samples were digested in concentrated nitric acid (HNO_3_) at 230 °C for 180 min and in perchloric acid (HClO_4_) at 310 °C for 20 min. They were then hydrolyzed in 9% hydrochloric acid (HCl) to reduce Se^6+^ to Se^4+^. Selenium was derivatized with 2,3-diaminaphthalene to form a selenediazole complex. A detailed method for the determination of selenium is presented in work by Skibniewska et al. [36]. The accuracy of the analytical method was based on NCS ZC 71001 reference material (bovine liver, n = 5; China National Analysis Center for Iron and Steel, Beijing, China). The selenium concentration in the tested gonads was 91% of the reference value. Analyses were performed in triplicate. Selenium content in the organs studied was expressed in mg·kg^−1^ wet weight.

### 2.3. Statistical Analysis

Statistical calculations were performed using the statistical package Statistica version 13.0 (TIBCO Inc.™ StatSoft, Krakow, Poland). The normality of the distribution of the variables was tested using the Shapiro–Wilk *W* test. Since the data did not have a normal distribution, the Mann–Whitney *U* test was used to compare differences between groups at a significance level of *p* ≤ 0.05 and *p* ≤ 0.01. Analysis of correlations between selected variables was performed using the Spearman correlation.

## 3. Results and Discussion

The basic physiological data of the animals studied are shown in Table 1.

Based on the results obtained, it was determined that the contents of selenium in the tissues of all animals tested ranged between 0.180 and 0.755 mg·kg^−1^ of wet-tissue weight and the median value for all 63 cats studied was 0.455 mg·kg^−1^ of wet-tissue weight. Results for young individuals and adult cats are presented in Table 2 and Table 3. However, it should be noted that males were represented by juvenile cats, as the median age in this group was only 8 months (Table 1). Healthy females were characterized by higher Se content compared to females with mammary gland carcinomas.

**Figure 1:**
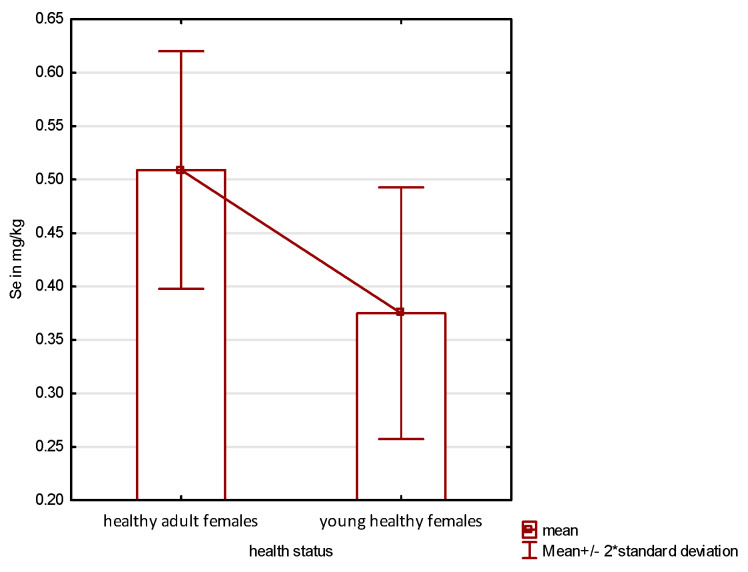
Se content in the ovaries of healthy females. Mean values in both groups are statistically significantly different.

The highest selenium content was registered in young males, in which the median was 0.226 mg·kg^−1^ higher than in the group of young females and 0.1 higher than in adult healthy females. Comparing healthy females, it was found that that there were statistically significant differences between both groups studied (Figure 1).

**Figure 2:**
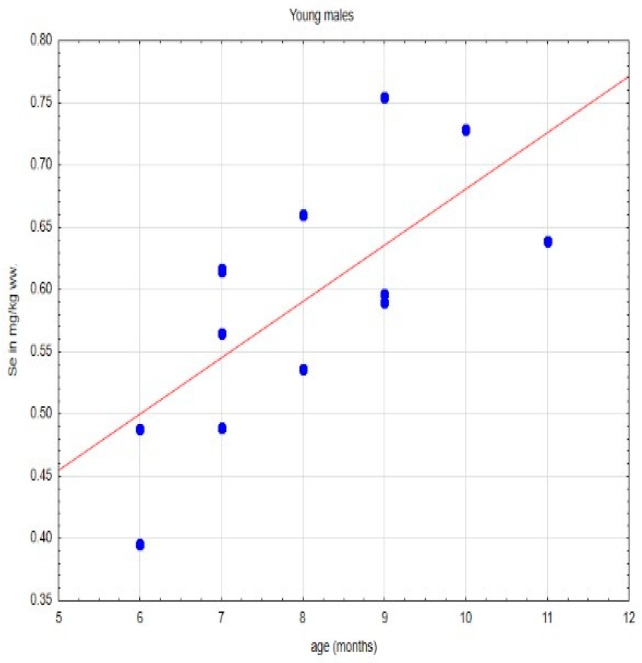
Se content in gonads of young males studied in relation to their age (age is expressed in months).

In the available literature on various animal species, long-term Se deficiency impairs the reproductive capacity of both males and females. This manifests itself, among others, in the form of a lower conception rate, increased embryonic mortality, low birth weight of offspring, and reduced growth rate of newborns, while causing retention of the placenta [6,37,38,39,40]. Selenium is also involved in embryo implantation and maintenance of normal placental function [8]. A too low Se content in a tissue can result in impaired steroidogenesis and hormonal regulation. Deficiency of this element can cause testicular degeneration, resulting in low sperm count and reduced sperm motility. Schomburg et al. [37] showed that in Se-deficient males, sperm count and fertility are significantly reduced. Even in cases where sperm motility is adequate, this peroxidation-induced DNA damage prevents fertilization of the ovum [37,41,42]. Administration of selenium to females during pregnancy was found to affect the growth efficiency of offspring regardless of postnatal management [43,44,45]. An adequate supply of selenium was also shown to positively affect hormonal profiles in females of various animal species. In adult rats during estrus, selenium supplementation significantly increases LH, FSH, estradiol, and progesterone, as well as prolactin concentrations [44]. In beef cows, selenium supplementation was found to increase progesterone levels throughout pregnancy and in the early luteal phase of estrus, while decreasing prolactin levels in late lactation [42]. In addition, Se deficiency in animals at birth and in adults disrupts bone metabolism, causing osteopenia and osteoarthritis [46,47,48,49]. As already mentioned, selenium is essential for the proper functioning of the immune system [50,51,52]. Its deficiency also affects aging processes, and many other metabolic processes [37,53,54]. Subclinical selenium deficiency causes less obvious effects and is recorded at the biochemical level [42,55,56]. Literature data indicate that in mammals, the testis is the organ where selenium accumulation necessary for proper spermatogenesis occurs [8]. In an analysis of the pharmacokinetics of Se-containing compounds, significant differences were found between its levels in the male and female gonads, as well as in the time of deposition of this element in the reproductive organs of individuals of both sexes. It was found that ovarian tissue stores it relatively quickly, although it does not reach the values recorded in male gonads, which was also confirmed by our research (Figure 1). The testes capture Se with some delay, however, it reaches much higher levels in their tissue than it does in females [57]. The available literature lacks data for cats; however, studies conducted on other species clearly indicate that Se content increases during the period of reaching sexual maturity, which is associated with gonadal function. It has been found to be essential for protecting sperm mitochondria from the effects of peroxidation of their structures [58]. Given that the process of castration of males mainly affects young individuals, it must be concluded that in the cats studied, the levels of Se in the testes have probably not yet reached the peak values found in individuals showing full sexual activity. Despite the fact that the median age in the study group was only 8 months, there were also sexually immature individuals aged 6 months. As is known from the literature, the period in which cats reach sexual maturity is subject to some fluctuation, but it usually occurs around 7–8 months of age [59,60]. Early castration in cats kept in urban conditions is a necessity. Due to the behavior of these animals, which, after reaching sexual maturity, begin to mark the area with urine, keeping them non-neutered is difficult to accept by owners. Therefore, the majority of indoor urban cats undergo gonadectomy before reaching sexual maturity. It is therefore to be expected that the level of Se in the testes of the male specimens studied would probably have increased even sooner. Unfortunately, there are virtually no data in the available literature on Se content in the gonads of cats, just as there are no studies on the requirement for this element, its metabolism and tolerance to various forms of selenium taken in the diet. There are reports that its concentration in blood serum in individuals of this species is up to five times higher than in other mammalian species [61,62,63]. Unfortunately, there is no information on the Se content of the testes of representatives of this species. Nevertheless, analysis of the data obtained in this study clearly indicates that the Se content in the testes of young males increases in the period up to maturity (Figure 2).

**Figure 3:**
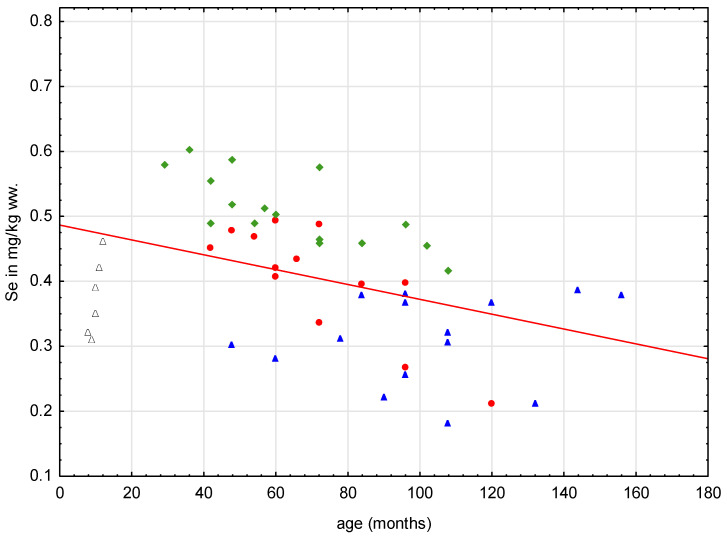
Distribution of Se content in all females studied according to their age. 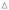 young females, 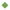 adult females, 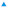 obese females, 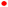 mammary carcinomas.

In the analysis of the effect of age on the content of Se in the gonads of the females studied, it can be seen that there is a clear difference between animals representing different age groups. This phenomenon is illustrated in Table 4. On tracking the Se concentrations in gonads of females, it can be seen that the highest values occur in mature females at the peak of their reproductive performance. All the values to the right of the boundary marked by 20 months of age, seen in Figure 3, are for females, in which the phenomenon of Se content in the ovaries decreasing with the age of the individuals studied is clearly visible.

There are few works on the role of Se in the reproductive processes of females, especially its contribution to ovarian function. As in males, in females Se plays a protective role against the effects of peroxidation of cellular structures and the phenomenon of oxidative stress. It is believed that reactive oxygen species produced by pre-ovulatory follicles are, together with hormonal activity, an important trigger for ovulation, while an imbalance between prooxidant and antioxidant levels causes ovarian dysfunction. Unfortunately, there are no studies of this nature in the domestic cat, and most of the work concerns women in whom low Se levels were accompanied by infertility and endometriosis [64,65]. Nonetheless, in this study, a clear trend associated with a decrease in Se content in ovarian tissue with the age of the animals is evident. In females over 10 years of age, the recorded contents do not exceed the value of 0.4 mg·kg^−1^ wet weight. The described phenomenon is confirmed by the analysis of the relationship between the age of the females studied and Se content, where a highly statistically significant negative correlation is observed between both studied factors (Table 5).

**Figure 4:**
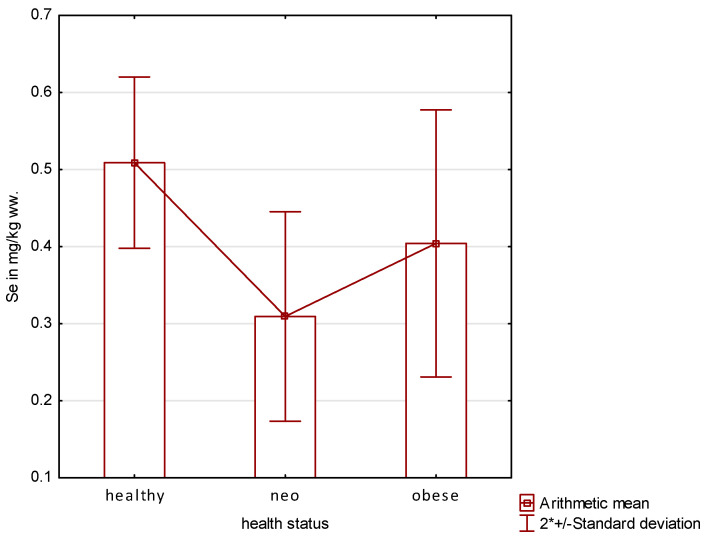
Se content in ovaries of adult females according to their health status. Mean values in each group are statistically significantly different at *p* ≤ 0.01.

When analyzing the effect of selenium status, it should be noted that there are highly statistically significant differences between selenium levels in the gonads of healthy mature females and obese cats and cats suffering from mammary gland carcinomas (Figure 4).

The median age of adult healthy females was 5.3 years, obese females almost 6 years, while cats with cancer were about 8.5 years old. The described groups can be arranged in the following descending order: healthy mature cats, obese cats, and cats with mammary gland cancer, in which a particularly low average Se content in the ovaries was registered. This is an interesting phenomenon, because it can be assumed that there is a connection between cancer disease and low Se content. The state of research to date indicates that an adequate supply of Se to the body is important from the point of view of preventing tumorigenesis. The anticancer effects of this element, or rather the selenoproteins, are associated with the inhibition of copper-dependent DNA damage, inhibition of peroxidation of cellular structures, and stimulation of apoptosis. In addition, they play an important role in the normal function of the immune system [18]. However, these are generally known effects of selenoproteins, which constitute an active antioxidant mechanism in all mammals of both sexes, while the exact mechanisms involved in the activity of mammary gland tissue and ovaries in terms of the effect of Se on their hormonal activity remain unknown to date. An additional difficulty in interpreting the results obtained is that studies of this nature have not been conducted in carnivorous animals, in which the ovarian cycle has characteristics different from those observed in laboratory animals or humans. Available results refer either to polyestrous laboratory animals, isolated cell lines derived from carnivores, or observations resulting from studies conducted in women in whom pathologies of the mammary gland or ovaries have been detected [18,25,66]. In view of the protective role of Se as an antitumor agent in various tissues of the body, an effective marker of its content in the body is currently being sought. It was found that this role can be played by glutathione peroxidase (GPx), hence, it is now being determined as a marker of the body’s selenium status. However, it should be noted that it has not been universally recognized as a reference element fulfilling the role of the so-called “gold standard” [67,68,69,70]. Se is also commonly determined in blood serum. The selenium status of carnivores is known to change during cancer. Enginler et al. [71] found that dogs after surgical treatment of malignant mammary tumors of epithelial origin had significantly lower serum Se concentrations of 18.63 µg/dL on day 15 after surgery, compared to a control group of healthy animals with a Se content of 42.24 µg/dL. Pilarczyk et al. [72] found that dogs burdened with malignant tumors had a significantly lower serum Se content than healthy individuals. In diseased animals, the content ranged from 103 to 265 µg/L while in healthy dogs, the recorded values ranged from 208 to 346 µg/L. Nevertheless, the authors concluded that the study should be continued to confirm whether this is a regularity that always applies to individuals with cancer. In this case, it is difficult to say whether the lower Se content in the serum of diseased animals is a result of the cancer or its cause. This can result, for example, from peroxidation of cellular structures, DNA damage, and poor immune system function, which can lead, as a consequence, to the development of cells with an abnormal phenotype. In this study, highly statistically significant differences (*p* ≤ 0.01) were found between individuals with cancer and healthy mature females, and the difference in mean values between the two groups was nearly 1.6 times in favor of healthy cats (Table 3). It is known that ovarian hormonal activity in cats, similar to other mammalian species, plays a role in mammary gland function. To date, it has not been conclusively demonstrated whether estrogen or perhaps progesterone has the ability to stimulate tumorigenesis in the mammary gland of cats, acting through the same molecular mechanisms seen in other species. However, it is known that feline mammary gland tumors have a lower concentration of estrogen receptors compared to canine or human mammary tumors. Hence, it has not been established whether estrogen, progesterone, or perhaps a combination of both hormones play an important role in the development of mammary gland cancer in this species. What is known, however, is that in the population of unsterilized female domestic cats, mammary gland carcinomas are seven times more common than in sterilized individuals, demonstrating their dependence on sex hormones produced by the ovaries [73]. Low Se content in the ovaries accompanying cancer of the individuals studied may be a cause of hormonal homeostasis disorders, exacerbating or initiating the tumorigenesis process in the mammary gland. However, it should be stated that there is no database of reference values for Se content in the ovaries of healthy cats. Forte et al. [74], investigating the Se content of ovaries of cats from two different habitats in Sardinia, found 1562 and 1617 ng·g^−1^ dry weight, respectively. After applying the conversion to mg·kg^−1^ wet weight, assuming that the ovarian tissue is approximately 70% hydrated, these values are 0.469 and 0.485 mg·kg^−1^ wet weight, respectively. It should be noted, however, that they studied young free-living females, while the cats analyzed in this study were well-fed companion animals of varying ages and health, kept in their owners’ homes. Forte et al. [74] found that in individuals less than 1 year old, the average Se contents of ovarian tissue, depending on the region, were 1652 and 817 ng·g^−1^ dry weight, respectively, while in animals aged 2–5 years, the values were 1559 and 940 ng·g^−1^ dry weight of the organ, respectively. After conversion, the values presented are 0.49, 0.24, 0.467, and 0.282 mg·kg^−1^ wet weight, respectively. The authors of the present study had a limited number of ovaries of females less than a year old in which the mean value was 0.375 mg·kg^−1^, while in healthy, non-obese females older than 20 months, the average Se content in ovarian tissue was 0.509 mg·kg^−1^ wet weight. It seems interesting to note the lower Se content in obese individuals, in which the average Se content was 0.404 mg·kg^−1^ wet weight of ovarian tissue, differing significantly from the average value recorded in healthy individuals. It is known that excess adipose tissue is responsible for the development of chronic inflammation and oxidative stress, which are important factors in other pathologies associated with obesity [75,76,77]. This phenomenon is associated with the activity of adipose tissue capable of releasing adipocytokines that cause the appearance of an inflammatory response in the body [78]. It is therefore to be expected that there is an increasing demand from the body for factors involved in protection against peroxidation, including selenium, because without their participation there is no possibility of effective control and reduction of the effects of cellular oxidation [79,80]. Studies in recent years found that Se is involved in the modulation of metabolic pathways in adipose tissue, such as adipogenesis, lipogenesis, lipolysis, oxidative stress, and finally the inflammatory response. Hence, its deficiencies may be involved in abnormal fat metabolism [81,82]. The findings cited above indicate that lower Se levels in obese cats than in healthy animals caused abnormalities in fat metabolism leading to excessive fat accumulation.

## 4. Conclusions

The Se content in the gonads of cats, as in other mammalian species, depends on the sex of the animals, as significantly higher levels of Se are observed in males compared to females. In adult females, a clear correlation is seen between Se levels in the ovaries and the animals’ health status. Obesity and mammary tumors are accompanied by low levels of Se compared to the values observed in clinically healthy animals. A marked decrease in Se content with the age of animals may be the cause of increased cancer incidence in older animals due to the weakening of mechanisms that protect against the effects of peroxidation of cellular structures and immune system function. Research on selenium status in carnivores should be continued in order to create a database of reference values to allow interpretation of the results obtained.

## Figures and Tables

**Table 1:** Characteristics of physiological data of the cats studied.

Group		N	Mean	SD	Median	Min.	Max.	Q_25_	Q_75_
Young males	Body mass	13	2.98	0.41	3.0	2.1	3.6	2.8	3.2
	Age		8.0	1.53	8.0	6.0	11	7	9
Young females	Body mass	6	1.83	0.26	1.8	1.5	2.2	1.6	2.0
	Age		10	1.41	10	8.0	12.0	9.0	11.0
Healthy females	Body mass	16	4.1	1.22	4.0	2.9	6.0	3.3	4.45
	Age		63.87	24.01	58.5	29.0	108.0	45.0	78.0
Obese females	Body mass	13	7.0	1.3	7.0	4.6	9.0	6.5	7.7
	Age		71.5	22.1	66.0	42.0	120.0	60.0	84
Females with mammary carcinomas	Body mass	15	3.91	0.68	4.0	2.8	5.0	3.4	4.5
	Age		101.6	29.08	96.0	48.0	156.0	84.0	120.0

Age is expressed in months, body mass in kilograms. Q_25_—lower quartile; Q_75_—upper quartile. SD—standard deviation.

**Table 2:** Selenium content in gonads of young males (in mg·kg^−1^ wet weight).

Group	N	Median	Min.	Max.	Q_25_	Q_75_
Healthy young males	13	0.5960	0.3950	0.7550	0.5360	0.6390

Q_25_—lower quartile; Q_75_—upper quartile.

**Table 3:** Selenium content in females studied (in mg·kg^−1^ wet weight).

Group	N	Median	Min.	Max.	Q_25_	Q_75_
All females studied	50	0.4120	0.1810	0.6020	0.3200	0.4870
Healthy young females	6	0.3700 ^A^	0.3100	0.460	0.3200	0.4200
Healthy adult females	16	0.4915 ^B^	0.4170	0.6020	0.4615	0.5650
Obese females	13	0.4200 ^C^	0.2120	0.5030	0.3950	0.4680
Females with mammary gland carcinomas	15	0.3120 ^A^	0.1810	0.3850	0.2560	0.3780

^A,B,C^—statistically significant differences at *p* ≤ 0.01 Q_25_—lower quartile; Q_75_—upper quartile.

**Table 4:** Selenium content in adult females representing different age groups (in mg·kg^−1^ wet weight).

Age group	N	Median	Min.	Max.	Q_25_	Q_75_
20–60 months	10	0.4725 ^A^	0.2800	0.6020	0.4200	0.5030
≥60–100 months	21	0.4330 ^AB^	0.2200	0.5880	0.3670	0.4890
≥100 months	13	0.3660 ^B^	0.1810	0.4640	0.3050	0.4170

^A,B^—statistically significant differences at *p* ≤ 0.05; Q_25_—lower quartile; Q_75_—upper quartile.

**Table 5:** Correlation of Se content in adult females in relation to their age and body weight.

	Se	Body Mass	Age (Months)
Se	------	−0.0668	−0.6753 **
Body mass	−0.0668	------	0.1427
Age (months)	−0.6753 **	0.1427	-------

** statistically significant differences at *p* ≤ 0.01.

## Data Availability

All data generated or analyzed during the study are included in this published article. The datasets used and/or analyzed in the current study are available from the corresponding author upon reasonable request.
